# Employment outcomes and job satisfaction of international public health professionals: What lessons for public health and COVID‐19 pandemic preparedness? Employment outcomes of public health graduates

**DOI:** 10.1002/hpm.3140

**Published:** 2021-04-04

**Authors:** Goel Treviño‐Reyna, Katarzyna Czabanowska, Sharmi Haque, Christine M. Plepys, Laura Magaña, John Middleton

**Affiliations:** ^1^ Department of International Health Care and Public Health Research Institute CAPHRI Faculty of Health Medicine and Life Sciences Maastricht University Maastricht The Netherlands; ^2^ Department of Health Policy Management Institute of Public Health Faculty of Health Sciences Jagiellonian University Krakow Poland; ^3^ Association of Schools and Programs of Public Health Washington District of Columbia USA; ^4^ Association of Schools of Public Health in the European Region (ASPHER) Brussels Belgium

**Keywords:** COVID‐19 pandemic preparedness, employment outcomes, job satisfaction, public health graduates, public health workforce

## Abstract

The profile of public health professionals (PHPs) and COVID‐19 preparedness is assessed against the employment outcomes (EO), precarious employment (PE), and job satisfaction (JS) of the European Public Health Master programme alumni. The study is descriptive, cross‐sectional, conducted from May‐October 2020. A survey was developed to assess the EO, PE and JS. Participants were recruited by email. SPSS statistics 26 version was used to perform descriptive analysis. A total of 189 PHPs participated (65% response) with majority women (66%), the mean age was 36 years. Participants were employed (80%), in non‐governmental organisations (20%), and academia (19%). Common employment positions were managerial (37%) and consultancy (18%). Majority of PHPs were exposed to PE (81%), the most frequent elements were ‘temporary employment’ (54%), and ‘the lack of labour union’ (53%). The JS of PHPs was ‘satisfied’. A blend of scientific public health knowledge and interpersonal competencies, reforms in current employment conditions, development of professional entities to safeguard PHPs' rights, and continuous investment in public health is necessary for PHPs to strengthen COVID‐19 pandemic preparedness. Furthermore, monitoring and evaluation of EO and JS are crucial to prepare PHPs according to the needs of the employment market and to be aware of PHPs' needs.

## 1 INTRODUCTION

The COVID‐19 pandemic declared by the World Health Organisation (WHO) is an example of the complex, diverse and interrelated nature of public health problems.^1^, ^2^ It is the political unwillingness that underpins these problems and social, economic and environmental factors further embed these challenges.^2^, ^3^ The public health workforce is unable to cope with the continuously changing needs of both current and future public health demands according to the WHO, the Association of Schools and Programs of Public Health (ASPPH) and the Association of Schools of Public Health in the European Region (ASPHER).^4^, ^5^, ^6^ The demand for qualified, competent Public Health Professionals (PHPs) to combat these challenges proves urgent.

A PHP is defined as an individual that engage in public health service practice irrespective of location but more specifically on educational background, specialized by means of academic bachelor, master's in public health or doctorate.^7^

Public health programmes have sharply grown in popularity particularly in the USA.^8^ Europe has mirrored a similar trend evidencing a 160% increase in the curricula validation of public health programmes and institutions in Europe (2017–2020 vs. 2014–2016).^9^ Its increasing popularity encompasses a variety of reasons, for example, it attracts individuals from diverse interdisciplinary backgrounds interested in making a global and local impact on the population's health, this is facilitated by the different options of public health curricula offered, from classic to novel paths.^10^ However, it seems that the pipeline between graduates and employments is misaligned, making it difficult for graduates to be able to secure first time employment in traditional public health sectors and being forced to enter positions outside of these roles.^10^, ^11^

The European Union Erasmus Mundus European Public Health master's (Europubhealth) programme, a recipient of annual funding from the European Commission is a part of the Erasmus Mundus Joint Diploma Master exemplifies the advocacy of both excellence and scholarship widening access to public health.^12^, ^13^ The Europubhealth offers a double master's in public health degree which diversifies specialisation options from the classic public health pathways such as epidemiology to novel ones such as leadership and governance, delivered by European universities.^14^

Public health system research has shown that the public health workforce operates with minimal resources characterised by lack of continuing education, shortage of professional staff, low wages, and a lack of professional organisations to safeguard employment rights.^2^, ^5^, ^10^, ^15^, ^16^ Working conditions are concordant with the definition of precarious employment (PE), an atypical and low‐quality employment.

PE depicts three features which include job insecurity, lack of social rights and protection, and income inadequacy.^17^ The dimension of employment insecurity is further divided into the next elements: temporary employment contract, multiple jobs, uncertainty of contract renewal.^17^ Lack of social rights and protection is further characterised into limited or no social benefits available (e.g., sick leave, bereavement leave, parental leave), limited or lack of working rights (e.g., protection against unfair dismissal, protection from authoritarian treatment, protection against discrimination or sexual harassment), and lack of representation (e.g., limited or no availability of labour or trade unions).^17^ Furthermore, PE has been linked with occupational injuries, adverse effects on mental, physical health and wellbeing.^17^, ^18^, ^19^, ^20^ PE fosters working conditions, which influence the level of job satisfaction (JS), referring to an individual's feelings about their job, directly affecting productivity, turnover, and physical and mental health.^21^, ^22^, ^23^, ^24^, ^25^ When present, these situations (PE and job dissatisfaction) may cause an exponentially negative effect on the PHPs, affecting their wellness and capacity to cope with the public health challenges.

The COVID‐ 19 pandemic has illustrated that no single nation is adequately prepared, exposing the gaps in the infrastructure of public health systems worldwide.^26^ These gaps endanger the success of PHPs to deliver public health functions, as they are not solely dependent on their competencies but based on both availability and conditions of their work. Employment outcomes (EOs) can help to define the profile of a PHP, for example, by including information about employment sector, job titles and place of work. Despite this, there is scarce information on EOs, the existence of PE on PHP's jobs is currently unknown, and the JS has been poorly addressed.^27^, ^28^

This study aims to contribute to describing the profile of PHPs, assessing the EOs, PE, and JS of PHPs who graduated from the Europubhealth programme. Furthermore, our results assess PHPs self‐reported perception of the adequacy of training received during the studies to participate in the COVID‐19 pandemic response.

### 1.1 Methods

The study was conducted from May to October 2020, in Maastricht, The Netherlands. An explorative, descriptive, and cross‐sectional design was used.

#### 1.1.1 Instruments

Data were obtained using a self‐developed questionnaire based on the questions related to graduate employment from the study conducted by the Association of Schools and Programmes of Public Health (ASPPH),^27^ and the fundamental concepts of PE: employment insecurity, income inadequacy, and lack of social rights and protection.^17^

The questionnaire comprised 35 questions on demographics, education, EOs, PE, and work during the COVID‐19 pandemic. The Minnesota Satisfaction Questionnaire (MSQ) short‐form was utilised to assess JS.^29^ It is an international validated survey that assesses general JS, as well as intrinsic (contextual factors around the work) and extrinsic factors (the content and effect of the work itself).^29^ The MSQ consisted of 20 items, each scored on a 5‐point Likert scale with 1 denoting strongly dissatisfaction, and 5 denoting strong satisfaction.^29^ The overall JS was estimated summing all item scores with scores from 61 to 80 considered satisfied, and scores from 81 to 100 considered very satisfied.

Descriptive questions were located first, followed by the JS questionnaire, and then questions regarding employment conditions. Further details about the questionnaire and the MSQ‐short form are found in Appendix 1 and 2.

An expert committee formed by four academics, PHPs with 25 to 30 years of experience in the field was consulted to review the final instrument for content validity and adaptation to the Europubhealth programme context. The final version was piloted with PHPs (*n* = 8) to guarantee correct format, language, sequence, and comprehension of the questions, and to estimate the duration to complete, no modifications were necessary.

#### 1.1.2 Study population

The whole Europubhealth alumni cohorts covering the years from 2006 (first generation) to 2019, were invited to participate in the online survey. The open‐access Europubhealth alumni directory was used to contact the potential participants.^30^ Further details about the Europubhealth programme are provided in Appendix 3.

#### 1.1.3 Data collection and analysis

A general invitation was posted on the social media groups of Europubhealth alumni to raise awareness about the survey. Using Qualtrics software, an invitation to participate in the self‐administered survey was sent by e‐mail. The objectives of the research and planned data management were explained in the invitation letter, all participants were assured of confidentiality and anonymity when they gave consent to participate. To recruit as many participants as possible e‐mail surveys and reminders were sent in weekdays and weekends, on the morning and afternoon trying to capture different participants' time zones and schedules. Reminders to participate were sent at weekly intervals to non‐respondents. When e‐mails were not successful, the researcher tried to contact participants through social media and/or peers.

Given the social science scope, 50% is deemed to be an average response rate.^31^

Four participants opted‐out and 23 did not complete the survey, thus, their answers were eliminated from the analysis. Available data on the numbers of alumni by generation was compared with the results with mean generational participation rate of 66%.^32^

A thematic analysis of the employment sectors and job titles based on frequency was conducted by Microsoft Excel. The Statistical Package for Social Sciences (SPSS) version 26 was used to perform descriptive statistics.^33^

#### 1.1.4 Ethical approval

This study was reviewed and approved by two members of the sub‐board panel of the Ethics Review Committee Health, Medicine and Life Sciences (FHML‐REC) of Maastricht University, The Netherlands.

## 2 RESULTS

The results of this study present the profile of PHPs, who graduated from the Europubhealth programme. The main findings encircle the following themes: general findings (participation and demographics), EOs, PE, JS, and training and participation of PHPs in the COVID‐19 response.

### 2.1 General findings

A total of 189 PHPs completed the survey. The response rate was 65%. The mean number of years since graduation was 6 ± 3.81 SD (minimum 1, maximum 12). On average, 66% of each cohort participated. Detailed information can be found in Appendix 4.

The profile of a PHP encompasses a young professional (79% < 41 years old), predominantly female (66%), who holds a bachelor related to human health on the areas of medicine, psychology, nursing, nutrition, dentistry, or physiotherapy (42%), most specialized in epidemiology, biostatistics, health economics, and environmental health (62%), and completed or continuing education after finalizing Europubhealth (39%). Detailed education information can be found in Appendix 5.

### 2.2 Employment outcomes

**FIGURE 1:**
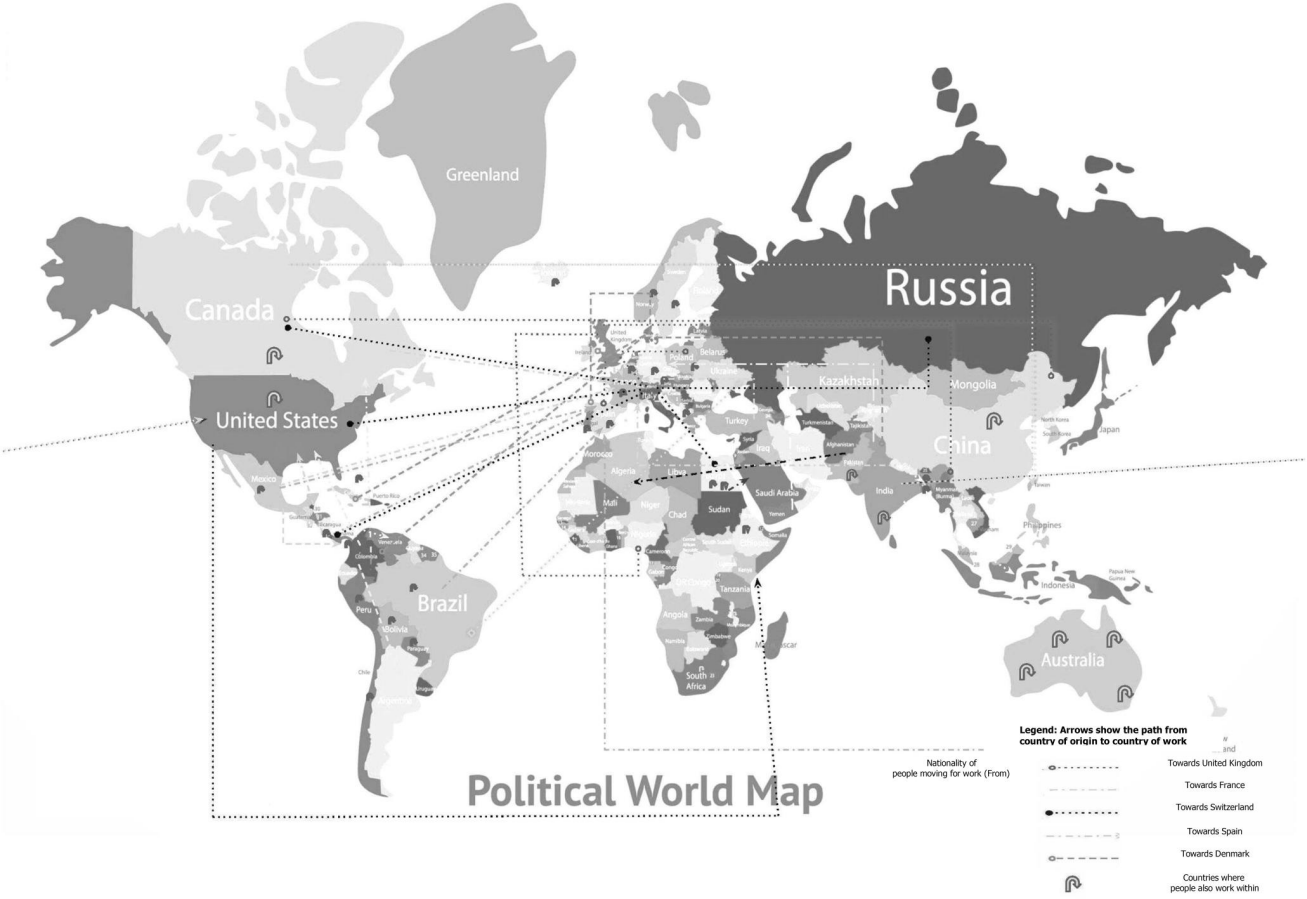
The World Map displaying the mobility of public health professionals (PHPs) from country of origin to country of work

The respondents represent a total of 61 nationalities from all continents, 38 % of PHPs were working in a different country from their country of origin (Figure 1), being the major host countries The United Kingdom (26%), Spain (12%) and France (11%). Detailed information of nationalities and employment mobility can be found in Appendix 6 and 7.

The majority of the PHPs were employed (80%), followed by those who were seeking employment or seeking continuing education (7%), those who were following continuing education, being part of a training programme or volunteering summed up a 11%, and those not employed nor seeking employment (2%). The mean length in current employment was 3 years ± 40.23 SD (minimum 1 month, maximum 20 years). More than half (61%) of PHPs found their first public health‐related job in less than 6 months, and 77% found their first job within one year. At the time of the survey, 5% of the PHPs were still looking for a job with a mean of 11 months ± 5.55 SD (minimum 4, maximum 22). The PHPs who did not find a public health‐related job and decided to start working in another field were looking for a public health‐related job during a mean of 16 months ± 18 SD (minimum 3, maximum 48) before deciding on employment in another area.

### 2.3 Employment market

Most PHPs had filled vacancies in the health or public health workforce at the time of the survey (95%), 80% identified their job as public health related, and 5% of PHPs were not working either in health‐related or public health‐related jobs. The majority were employed in not‐for‐profit (78%).

The entities employing PHPs were identified in seven sectors: non‐governmental organizations (20%), academia (19%), government (19%), healthcare (16%), business (15%), intergovernmental organizations (8%), and self‐employed (3%; Table 1). Names of the entities per sector can be consulted in Appendix 8.

**TABLE 1:** Employment outcomes of public health professionals (PHPs) by job positions and job sectors (*N* = 152)

Job position/sector	Academia	Business, industrial, commercial firm	Government	Healthcare	Non‐governmental organisations	Intergovernmental organisation	Self‐Employed	Total
Manager	3	10	11	6	15	5	1	51 (34%)
Consultant		3	6	2	8	6	1	26 (17%)
Researcher	11	2	2	2	2			19 (13%)
Medical personnel				10				10 (7%)
Academic	10							10 (7%)
Coordinator	1		2	1	2			6 (4%)
Analyst		2			1	1		4 (3%)
Epidemiologist	1		2	1				4 (3%)
Statistician			1	1				2 (1%)
Other	1	5	3	1	2		2	14 (9%)
Missing	2	1	2		1			6 (4%)
Total	29 (19%)	23 (15%)	29 (19%)	24 (16%)	31 (20%)	12 (8%)	4 (3%)	152 (100%)

The job positions were organized into five areas: manager (34%), consultant (17%), researcher (13%), medical health personnel (7%), academic (7%), coordinator (4%), analyst (3%), epidemiologist (3%), statistician (1%), and others (9%; Table 1). Job titles can be consulted in Appendix 9.

Further analysis concerning the PE, JS and participation in the COVID19 pandemic responses only includes PHPs who were employed in health or public health‐related areas (*n* = 147).

### 2.4 Precarious employment

Two of the three dimension of PE were present in the PHPs' employments: job insecurity (60%), and lack of social protection and rights (55%). On the dimension of job insecurity, the most frequent element was the temporary work contract (self‐employed, fixed‐term, payment per project; 54%), and uncertainty about the next contract renewal (28%). In the dimension of lack of rights and protection, the lack of unions was present in more than half of the PHPs (53%), of those who had a union available, only 35% responded that they were part of it, being the 16% of the total population. All reported wages were above the lowest income quartile, which would mean that all the PHPs had adequate income (Table 2). The 81% of the PHPs experienced one dimension of PE, and 34% had two dimensions of PE in their jobs (job insecurity and lack of social protection and rights).

**TABLE 2:** Precarious employment of public health professionals (PHPs)' employed in health or public health‐related jobs (*N* = 147)

Dimension	*N* (%)	Element	*N* (%)
Job insecurity	88 (60%)	Temporary work contract	79 (54%)
		Multiple jobs	33 (22%)
		Uncertainty of contract renewal	41 (28%)
Lack of social protection and rights	81 (55%)	Lack of unions	78 (53%)
		Lack of access to social non‐wage employment benefits^a^	16 (11%)
		Lack of access and/or power to exercise workplace rights^b^	14 (10%)
Insufficient income	0 (0%)		0%

^a^ Such as sick leave, domestic leave, bereavement leave, or parental leave.

^b^ Such as protection against unfair dismissal, protection from authoritarian treatment, protection against discrimination, or protection against sexual harassment.

### 2.5 Job satisfaction

Results of the Minnesota questionnaire showed that in general the PHPs were ‘satisfied’ with their job with 50% of PHPs ‘very satisfied’, 42% ‘satisfied’, and 8% ‘neutral’. The items rated lowest were ‘The way company policies are put into practice’ and ‘The chances for advancement on this job,’ which were both extrinsic factors. Detailed information of JS can be found in Appendix 10.

Participation in the COVID‐19 pandemic response.

The 70% percent of PHPs considered having adequate training to participate in the COVID‐19 pandemic response, and from this percentage, 65% were participating in the response. From the entire population, 57% of PHPs were involved somehow in the response of the COVID‐19 pandemic and 63% of those who did not, expressed their willingness to be involved.

## 3 DISCUSSION

The study presents the profile of a PHP being a young professional, predominantly female, specialised in classic public health disciplines, following continuing education, with diverse backgrounds mostly related to human health, representing various nationalities and with a high level of international job mobility. Most of the PHPs are employed in public health and health related jobs located in six sectors, sharing five main employment titles among them. Two of the three dimensions of PE are present in the employment of PHPs, and yet PHPs are satisfied with their job. Slightly more than half of the PHPs participate in the COVID‐19 response with others expressing their willingness to do so. A relatively high percentage of PHPs report having adequate training to respond to COVID19 pandemic.

Our findings show that common specialization paths are still the classical ones: epidemiology and biostatistics. This mirrors a prevalence of traditional public health pathways which is also documented in the USA.^27^ However, our findings demonstrate that the most prevalent employers are non‐governmental organizations and academia, and the most frequent job positions for PHPs are managerial and consultancy. Our results are concordant with research that analysed global health vacancies gathered from twelve internet job boards based in the USA, where the most common skill areas were management (36%) and technical expertise (20%).^34^ Our findings are also in the line with the literature review from the USA on master's public health graduates which included 11 public health schools, that documented a flat governmental hiring rate of 12.53% in average during the five years included (2012–2016) despite of the increment of PHPs graduated in those years.^11^ These findings support the theory that governmental bodies are no longer the sole principal recruiting institutions; private companies now represent a significant part of the employment sector for PHPs,^11^, ^35^ suggesting greater acceptance and recognition of public health amongst the population that it serves.

Both managerial and consultancy areas need a set of skills that rarely can be obtained from typical public health education alone.^36^ These areas require years of expertise and are not entry level jobs, making it difficult for graduates to find a job in the public health employment market.^34^ Since a decade ago the mismatch between the educational pipeline and public health employment was raising concerns due to the outpacing supply of graduates that were forcing PHPs to find jobs outside of the public health area.^37^ Although there are studies that forecast the public health workforce shortage, specific details on worker's discipline, training level, and functional ability remains unclear.^38^ Public health workforce crisis has been exacerbated by poor recruitment and retention strategies causing harmful impact on population health and its systems.^39^ Certainly, a continued assessment of EOs of graduates is needed to increase awareness in the current trends of employment and to give opportunity to improve the academic curricula with the aim to prepare PHPs with the competencies needed in the public health employment market.

Contributing factors such as the existence of PE make the employment market for PHPs even more challenging. The presence of employment insecurity (60%), and lack of social rights and protection (55%) in our population were evidently high. Temporary employment (employment insecurity) limits the capacities of PHPs to deliver quality competencies, typically temporary workers get fewer professional trainings and opportunities to develop their careers.^40^

While the lack of labour unions already puts PHPs in a vulnerable position, unable to demand their lawfully benefits and rights, professional chambers are documented to be largely non‐existent for PHPs.^41^, ^42^ Where professional chambers exist, they contribute to safeguard the rights and privileges of the represented professionals, for example, by formally providing license and credentials, setting standards for education, skill levels and competences, and serve the profession in a sense of a collective voice.^41^, ^43^ A precursor of a PHP chamber can be the Faculty Public Health (FPH) in the UK with national professional regulation, for example, devising the curriculum for specialist public health training and being part of the three Royal Colleges of Physicians in the UK.^42^ Labour unions are expected to manage both collective bargains and agreements in the workplace, regulate wages and working conditions covering all employees regardless if they are members or not of a union.^44^ Our study results highlighted that professionalisation of the public workforce and the absence of professional chambers had played a role in EOs for PHPs.^11^

Despite the fact that 81% of our population had one dimension of PE, and 34% had two of three dimensions of PE in their jobs, our population was satisfied with their job. Our results echo the theory that argues that in some specific cases, such as in highly skilled workers, when the non‐standardized employment conditions are chosen voluntarily, it can enhance JS and quality of life.^45^ Besides, our results showed lower scores on the extrinsic area, studies suggest that this is an area of opportunity as particularly extrinsic factors are easy to improve and thus help both productivity and job retentions of PHP.^34^

JS is one of the key drivers for the mobility of PHPs.^46^ The nature of the Europubhealth programme could be driving PHPs mobility by supporting professional networking and international professional experiences. As with other Erasmus Mundus programmes, Europubhealth provides the opportunity to study and live in at least three different European countries. In addition, during the last month of the academic programme, students have the possibility to select another country (worldwide) for professional practice and final thesis.^13^ Drawing upon the findings, it was reported that 38% of participants were employed in a different country to that of origin. PHPs mobility to other countries can provide both better health and access to healthcare opportunities to the host populations, although there is also danger of contributing to the well‐known brain drain.^15^

The mobility of PHPs from the place of origin has negative consequences as there is a loss of PHPs who are unable to participate in public health responses particularly in times of crisis.^7^ Motivators to move to other countries include poor remuneration, poor working conditions, unstable and oppressive political climate and discrimination.^47^ On a career frontier, PHPs face a limited career structure and poor intellectual stimulation.^48^ Developing nations may not be able to harness the full potential of PHPs of their home country and it is exacerbated by lack of funding and poor facilities.

Our findings seem to reveal some of the weaknesses of the public health system and its preparedness in times of COVID‐19 pandemic: mismatches between education and employment market, presence of PE and mobility of PHPs. However, a bold percentage of PHPs (43%) were not participating remains. Further research could explore the reasons behind this phenomenon to understand better the reasons PHPs are not participating in pandemic response.

The COVID‐19 outbreak has illustrated the obstacles that public health faces where the workforce emerges as largely outside the medical profession yet still within the biomedical model.^49^ In order to bolster future pandemic preparedness, public health roles should expect further investment in all areas, including basic and continued education, research to better understand the current situation and needs of the employment market, as well as to assure that the objectives of academic and social programmes are reached.^37^ A strong advocacy of a clear competency‐based recruitment, personnel and human resource system need to be implemented in order to reform both hiring practices and related salary structures.^38^ The COVID‐19 pandemic has reinforced the wider need in public health workforce of a plethora of skills including economic evaluation, behavioural psychology, social investigation in the field of inequality, healthy public policy, environmental science and protection, and community development. Furthermore, the figure of a PHP as an expert on public health needs to be fostered to create the sense of leadership and expertise for future health challenges, improving the trust and communication between experts and population, giving more weigh to the knowledge of the PHPs as experts on the public health area.^50^

### 3.1 Limitations

This study explores a specific population of Europubhealth alumni; individuals with a wide range of backgrounds and nationalities, more than half of them awarded with academic excellence scholarship, which frames their academic prowess. The selective nature of the programme may influence their EOs in public health. However, caution must be placed as this study is not representative of all PHPs and limited research is available.

The method executed in this study was a self‐reported questionnaire exposing possibility of bias.^32^ To diminish the social desirability bias, the objectives of the research and planned data management were explained in the invitation letter, full anonymity was provided, force‐choice items were present and a self‐administrate instrument was used. To decrease the order bias, descriptive questions were located first, followed by the JS questionnaire, and lastly, questions regarding employment conditions. Nonresponse bias was another potential limitation of this study. It was counteracted by trying to get as many participants as possible; e‐mail surveys and reminders were sent in weekdays and weekends, in the morning and afternoon trying to catch different participants' time zones and schedules, a general invitation was posted on the social media groups of Europubhealth alumni to raise awareness about the survey. Non‐responders were contacted to offer support to complete the survey; available data on the numbers of alumni by generation was compared with the results, having a mean generational participation rate of 66%.^32^ Finally, recall bias could be present as some of the questions were related to events in the past.^32^

## 4 CONCLUSIONS

This study documents a relatively high employment rate among PHPs who graduated from the EU Erasmus Mundus Europubhealth, which contributes to global job mobility of professionals. Academic programmes such as Europubhealth can prepare PHPs with competencies that assist and prepare PHPs for public health challenges such as the COVID‐19 pandemic. Such programmes should combine scientific evidence based public health knowledge and interpersonal competencies, including communication, response, and preparedness. These competencies also fit with the needs of the current employment market, as they are expected for managerial and consultancy positions.

This group of PHPs is satisfied with their job, but their employment situation is unstable due to employment insecurity and lack of professional protection entities to safeguard their employment rights. It is necessary to continue the monitoring and evaluation of EOs of PHPs to identify the evolving job market needs and to prepare the graduates of public health programmes to address adverse situations. It is necessary also to continue to lobby for increasing professional recognition and standards and for formal professionalisation of the public health workforce.

Strengthening preparedness for the COVID‐19 pandemic, requires significant and continuous investment in public health, provision of education that fits the needs of the public health challenges and the employment market, reforms of public health employment conditions, and development of professional entities to safeguard PHPs' rights.

## CONFLICT OF INTEREST

No conflict of interest for all authors.

## ETHICS STATEMENT

This study was reviewed and approved by two members of the sub‐board panel of the Ethics Review Committee Health, Medicine and Life Sciences (FHML‐REC) of Maastricht University.

## Data Availability

The data that support the findings of this study are available on request from the corresponding author. The data are not publicly available due to privacy or ethical restrictions.

## 1

The survey instrument was developed based on the common questions presented on graduate employment from the Association of Schools and Programs of Public Health (ASPPH),^27^ and the fundamental concepts of precarious employment: (1) Employment insecurity (temporal contract, multiple jobs, uncertainty of contract renewal); (2) Lack of social rights and protection, further divided into (a) Limited or no social benefits available (e.g., sick leave, bereavement leave, parental leave); (b) Limited or lack of working rights (e.g., protection against unfair dismissal, protection from authoritarian treatment, protection against discrimination or sexual harassment), and (c) Lack of representation (e.g., limited or no availability of labour or trade unions); and, (3) Income inadequacy (material deprivation).^17^

Questions 1–3 assess demographics, 4–9 education, 10–21 employment outcomes and employment conditions; questions 13, 14, 22–24 assess elements of employment insecurity, 25–28 assess the lack of rights and protection, and 29–32 assess elements of income inadequacy; questions 33–35 assessed the participation of PHPs on the COVID‐19 pandemic.

### 1.1 Part 1. Demographics and education

1. Please enter your year of birth.

Four‐digit year.

2 What is your sex?
  - Female
  - Male
3 What is your nationality?
  - List of countries
4 What did you study as a bachelor's degree?

Open ended question.

5 What was your first year of Europubhealth+?
  - List of options
6 What was your second year of Europubhealth+?
  - List of options
7 When did you graduate of Europubhealth+?
  Four‐digit year.

8 Did you completed other degree programme in public health or another field after Europubhealth+?
  - Yes, in PH.
  - Yes, in another field.
  - Yes, in both.
  - No
9 Are you currently continuing your education through enrolment in another degree programme in public health or another field?
  - Yes, in PH.
  - Yes, in another field.
  - No

### 1.2 Part 2. Employment outcomes

10 Were you employed while obtaining your Europubhealth + degree?
  - Yes, Full‐time (40 h per week, with a contract lasting >1 month)
  - Yes, Part‐time (<35 h per week, with a contract lasting >1 month)
  - Yes, temporary work (Including fixed‐term and subcontracted jobs, as well as work done on projects, on call and through temporary‐help agencies.)
  - Yes, self‐employed.
  - No

10.1. If yes, was your employment related to PH?
  - Yes
  - No
10.2. If yes, did you continue working in the same job after completion of EUROPUBHEALTH+?
  - Yes
  - No
10.3. if yes, were you employed in the same position prior to/concurrent to earning your Europubhealth + degree?
  - Yes
  - No

11 (if NO to question 10 or not related to PH) How long did it take you to get your FIRST Public Health related job/position (part‐time or full‐time) after completion of EUROPUBHEALTH+?
  - Less than 6 months
  - 6 months
  - 1 year
  - More than 1 year
  - I didn't find a job in PH after # months or years and start working in another field *open to add the time.
  - Not seeking/sought for a job in PH.
12 What is your current employment status?
  - Employed: Employed in a full‐time or part‐time position, temporary position, self‐employed (not fellowship, internship, postdoctoral, residency or volunteer position)
  - Training Programme Participant: Participating in a fellowship, post‐doctoral fellowship, internship, or residency.
  - Volunteer Participant: Participating in a volunteer or service programme (e.g., Peace Corps, mission work)
  - Continuing Education: Not employed and have been accepted to and plan to matriculate into a programme of further study or training.
  - Not Employed, Seeking Employment or Continuing Education: Not employed and seeking employment or engaged in the job search process, or seeking and have not enrolled in a programme of continuing education/training.
  - Not Employed, Not Seeking: Not employed and not pursuing either employment or continuing education at this time.

13 Do you have a second job?
  - Yes
  - No
14 Do you have a third job?
  - Yes
  - No
15 Which of the following best describes your primary employment sector?
  - Academic Institution: Includes elementary, secondary, or post‐secondary academic institution.
  - Government Agency: Includes Federal, State, Local, Tribal government agency, military.
  - Healthcare Organization: Includes hospital or healthcare provider, managed care organization, etc.
  - Business, Industrial, or Commercial Firm: Includes health insurance or health IT company; consulting firm; marketing, public relations, or communications firm; pharmaceutical, biotech, or medical device firm; or other industrial, commercial, or for‐profit firm.
  - NGO, association, foundation, voluntary.
  - Self‐employed
  - Other: open answer
16 Which sector does your current (main) employment/job come under?
  - for‐profit
  - no‐profit
  - I do not know
17 Where is your work located?
  - Countries
18 What is the name of the organization, firm, or company where you are employed?

Open‐ended response.

19 What is your job title?

Open‐ended response.

20 Do you consider your work health‐related?
  - Yes
  - No
21 Do you consider your work public health‐related?
  - Yes
  - No
22 How long have you been on your present job? ____ years _____ months.

### 1.3 Part 4. Employment conditions

23 What kind of contract do you hold with your current employer?
  - Fixed term (contract for a determined amount of time)
  - Temporal (work on projects, on call and through temporary‐help agencies.)
  - Permanent (contract for an unlimited duration)
  - Self‐employed
  - Other: open answer
24 Do you have certainty about the renewal of your next contract?
  - Yes
  - No
25 In your current employment, are there trade unions available for you?
  - Yes
  - No
26 Are you part of a trade union?
  - Yes
  - No
27 Do you have access to standard non‐wage employment benefits such as sick leave, domestic leave, bereavement leave or parental leave?
  - Yes
  - No
28 Do you have access and/or power to exercise workplace rights such as, protection against unfair dismissal, protection from authoritarian treatment, discrimination, or sexual harassment?
  - Yes
  - No

### 1.4 Part 5. Salary

29 Indicate your monthly base salary, please add the money currency used.

Monthly Base Salary: [Numerical]

30 Do you find this salary appropriate for your job responsibilities?
  - Yes
  - No
31 Based in your responsibilities, what would be a fair salary for you?

Monthly base salary: [Numerical], please add the money currency used.

32 How does this salary compare with your salary prior to receiving your Europubhealth + degree?
  - Less
  - Same
  - 1%–5% more
  - 6%–10% more
  - 11%–25% more
  - 26%–50% more
  - >50% more
  - Not applicable (Not employed prior to obtaining public health degree.)
33 Are you involved as a PH professional in the COVID‐19 pandemic?
  - Yes, principal function.
  - No
34 If not, would you like to be involved?
  - Yes
  - No
35 Do you think that you hold adequate training to support the PH response to the crisis caused by COVID‐19?
  - Yes
  - No

End of survey.

Thank you for the taking the time to complete this survey! Your responses will help to foster the professionalization of our career.

## APPENDIX 2 The Minnesota satisfaction questionnaire (short‐form)

### 2.1

Job satisfaction, the individual's feelings about their job, is the result of extrinsic and intrinsic factors (EF, IF); the EF refers to the contextual factors that are around the work; such as payment, type of contract, temporariness, job benefits, and job security, it also includes human relations as supervision, co‐workers, and personal life; the IF refers to the content and effect of the work itself, including the functions, responsibilities, relevance of the work, recognition, and advancement.^29^

The MSQ short‐form is a self‐administrated and international validated survey, consisting of 20 questions; questions 1–4, 7–11, 15, 16, 20 assess intrinsic factors; questions 5, 6, 12–14, 19 assess extrinsic factors, and all questions assess general job satisfaction.^29^

Each question has five response alternatives with stablished scoring weights: very satisfied 5, satisfied 4, neutral 3, dissatisfied 2, very dissatisfied 1.

To interpret the results, raw scores for the scales (general, intrinsic, extrinsic) were ranked as follows;

**Table:**

General satisfaction	Intrinsic factors	Extrinsic factors
0 to 20—very dissatisfied	0 to 12—very dissatisfied	0 to 6—very dissatisfied
21 to 40—dissatisfied	13 to 24—dissatisfied	7 to 12—dissatisfied
41 to 60—neutral	25 to 36—neutral	13 to 18—neutral
61 to 80—satisfied	37 to 48—satisfied	19 to 24—satisfied
81 to 100—very satisfied	49 to 60—very satisfied	25 to 30—very satisfied

On my present job, this is how I feel about	VS	S	N	D	VD
1. Being able to keep busy all the time					
2. The chance to work alone on the job					
3. The chance to do different things from time to time					
4. The chance to be ‘somebody’ in the community					
5. The way my boss handles his/her workers					
6. The competence of my supervisor in making decisions					
7. Being able to do things that do not go against my conscience					
8. The way my job provides for steady employment					
9. The chance to do things for other people					
10. The chance to tell people what to do					
11. The chance to do something that makes use of my abilities					
12. The way company policies are put into practice					
13. My pay and the amount of work I do					
14. The chances for advancement on this job					
15. The freedom to use my own judgment					
16. The chance to try my own methods of doing the job					
17. The working conditions					
18. The way my co‐workers get along with each other					
19. The praise I get for doing a good job					
20. The feeling of accomplishment I get from the job					

## APPENDIX 3 The Europubhealth programme

### 3.1

The Europubhealth programme consists of 2‐year multidisciplinary master course in public health funded in 2006. Graduates receive a double master's degree composed of a master's degree from their first‐year institution + a master's degree from their second‐year institution corresponding to the specialization.^14^.

- 1st year—foundation course, core competencies in Public Health.
  - University of Sheffield (UK), since 2006.
  - University of Granada (Spain), since 2006.
- 2dn year—specialization course
  - Maastricht University (The Netherlands)‐ Leadership in European Public Health, since 2017.
  - The Jagellonian University (Poland)—Governance of Health Systems in transition—Health Economics and Financial Management—Social and Health Protection, since 2006.
  - The School of Public Health (France)—Advance Biostatistics and Epidemiology—Environmental and Occupational health sciences, since 2006.
  - The University of Rennes (France)—Health Policies and Programmes Management—Law, Health and Ethics, since 2006.
  - University of Granada (Spain)—Health Services Management—Health promotion, since 2006.
  - Copenhagen—Quantitative assessment in Public Health—Advanced public health methods—Health Services and Prevention, from 2006 to 2017.

The Europubhealth programme is an Erasmus Mundus Joint Master, recognized as a Master of Excellence by the European Commission since 2006, and awarded the Best Practice Award of Excellence in Public Health Education and Training by ASPHER in 2018.^14^

The Europubhealth programme awards EU‐funded scholarships to the best student candidates applying under annual selection rounds.^12^, ^13^

### 3.2

**APPENDIX 4:** Participation and demographics of PHPs (*N* = 189).

Participation			Demographics	
Generation	Total alumni	Alumni participation (% per generation)	Age group	*N* (%)
2008	29	16 (55%)	25–30	39 (21%)
2009	37	19 (51%)	31–35	58 (31%)
2010	25	13 (52%)	36–40	51 (27%)
2011	28	12 (43%)	41–45	19 (10%)
2012	24	14 (58%)	46–50	14 (7%)
2013	17	12 (71%)	51+	8 (4%)
2014	22	14 (64%)	Sex	*N* (%)
2015	16	14 (88%)	
2016	12	8 (67%)	Female	125 (66%)
2017	24	18 (75%)		
2018	17	14 (82%)	Male	64 (34%)
2019	39	35 (90%)		
Total	290	189 (66%^a^)		

^a^ average participation per generation.

### 3.3

**APPENDIX 5:** Education of PHPs (*N* = 189).

Bachelor education *N* (%)					
Medicine	42 (22%)	Dentistry	3 (2%)	Arts	1 (0.5%)
Psychology	15 (8%)	Health education	3 (2%)	Ayurvedic medicine	1 (0.5%)
Public health	10 (5%)	Health service management	3 (2%)	Behavioural and community health	1 (0.5%)
Biology	8 (4%)	International relations	3 (2%)	Biochemistry	1 (0.5%)
Nursing	8 (4%)	Physiotherapy	3 (2%)	Chemical engineer	1 (0.5%)
Pharmacy	8 (4%)	Sports science	3 (2%)	Communications	1 (0.5%)
Health science	6 (3%)	Biotechnology	2 (1%)	Emergency management administration	1 (0.5%)
Nutrition	5 (3%)	Environmental health	2 (1%)	Equine science	1 (0.5%)
Social work	5 (3%)	International business	2 (1%)	European public health	1 (0.5%)
Anthropology	4(2%)	International studies	2 (1%)	Geographical engineering	1 (0.5%)
Economics	4(2%)	Microbiology	2 (1%)	History	1 (0.5%)
Politics	4(2%)	Veterinarian	2 (1%)	Latin American studies	1 (0.5%)
Sociology	4(2%)	Administration	1 (0.5%)	Life sciences	1 (0.5%)
Biomedical science	3 (2%)	Agrobusiness and resource management	1 (0.5%)	Molecular biology	1 (0.5%)
				Public administration	1 (0.5%)
				Missing	15 (8%)

1^st^ year Europubhealth institution	*N* (%)
University of Granada (Spain)	64 (34%)
University of Sheffield (UK)	125 (66%)

2^nd^ year institution and programme	*N* (%)
University of Copenhagen (Denmark)—Advanced methods in public health	37 (20%)
EHESP School of public health Paris (France)—Advanced biostatistics and epidemiology	32 (17%)
EHESP School of public health Paris (France)—environmental and occupational health	25 (13%)
Jagiellonian University in Krakow (Poland)—health economics and governance of health system	24 (12%)
University of Copenhagen (Denmark)—health services and prevention	23 (12%)
Jagiellonian University in Krakow (Poland)—social and health protection	17 (9%)
Jagiellonian University in Krakow (Poland)—health economics and Financial management	12 (6%)
Maastricht University (The Netherlands)—leadership in European public health	6 (3%)
Andalusian School of public health—University of Granada (Spain)—health promotion	3 (2%)
Jagiellonian University in Krakow (Poland)—governance of health system in transition	3 (2%)
University of Rennes 1/EHESP Rennes (France)—Droit, Santé, Ethique	3 (2%)
EHESP School of public health Rennes (France)—Pilotage des Politiques et Actions en Sante Publique	2 (1%)
Andalusian School of public health—University of Granada (Spain)—health management	1 (0.5%)
Andalusian School of public health—University of Granada (Spain)—quality improvement	1 (0.5%)

Continuing education after the Europubhealth degree	*N* (%)
Completed in public health	18 (10%)
Completed in another field	16 (9%)
Completed in both	7 (4%)
In progress on public health	16 (9%)
In progress in another field	14 (7%)
No	118 (61%)

### 3.4

**APPENDIX 6:** Nationalities of PHPs (*N* = 189).

Nationality *N* (%)							
The USA	30 (15.5%)	Bolivia	3 (2%)	Viet Nam	2 (1%)	Ireland	1 (0.5%)
Colombia	12 (6%)	Italy	3 (2%)	Yemen	2 (1%)	Malawi	1 (0.5%)
India	9 (4%)	Myanmar	3 (2%)	Afghanistan	1 (0.5%)	Panama	1 (0.5%)
Germany	8 (4%)	Netherlands	3 (2%)	Austria	1 (0.5%)	Paraguay	1 (0.5%)
Nepal	8 (4%)	Pakistan	3 (2%)	Belgium	1 (0.5%)	Russian Federation	1 (0.5%)
Spain	7 (3%)	Philippines	3 (2%)	Chad	1 (0.5%)	South Africa	1 (0.5%)
Brazil	5 (3%)	Poland	3 (2%)	Chile	1 (0.5%)	Sudan	1 (0.5%)
Egypt	5 (3%)	Serbia	3 (2%)	Cuba	1 (0.5%)	Sweden	1 (0.5%)
France	5 (3%)	Albania	2 (1%)	El Salvador	1 (0.5%)	Thailand	1 (0.5%)
The UK	5 (3%)	Bahamas	2 (1%)	Ethiopia	1 (0.5%)	Trinidad and Tobago	1 (0.5%)
Australia	4 (2%)	Bangladesh	2 (1%)	Finland	1 (0.5%)	Turkey	1 (0.5%)
Canada	4 (2%)	China	2 (1%)	Ghana	1 (0.5%)	Uganda	1 (0.5%)
Costa Rica	4 (2%)	New Zealand	2 (1%)	Guatemala	1 (0.5%)	Uruguay	1 (0.5%)
Mexico	4 (2%)	Nicaragua	2 (1%)	Hong Kong	1 (0.5%)		
Nigeria	4 (2%)	Norway	2 (1%)	Hungary	1 (0.5%)		
Peru	4 (2%)	Portugal	2 (1%)	Iceland	1 (0.5%)		

### 3.5

**APPENDIX 7:** Employment mobility of employed PHPs (*N* = 152).

With mobility 57 (38%)					
Nationality	Country of work	Nationality	Country of work	Nationality	Country of work
Afghanistan	Algeria	Cuba	Denmark	Nepal	The UK
Brazil	Ukraine	Egypt	Switzerland	New Zealand	Spain
Brazil	Spain	Egypt	United Arab Emirates	Nicaragua	Venezuela
Canada	Taiwan	Ghana	Sierra Leone	Nigeria	The UK
Canada	Switzerland	Hungary	The UK	Nigeria	The UK
Canada	The UK	India	Germany	Pakistan	Canada
Chad	France	India	Indonesia	Philippines	The UK
China	The UK	India	The USA	Poland	The UK
China	The UK	India	Denmark	Russian Federation	Switzerland
Colombia	Spain	Ireland	The UK	Serbia	France
Colombia	The USA	Italy	The UK	Spain	Denmark
Colombia	Argentina	Mexico	France	Spain	The UK
Colombia	Canada	Mexico	Panama	Turkey	France
Colombia	Canada	Mexico	Spain	The UK	France
Colombia	Denmark	Myanmar	The UK	The UK	Germany
Costa Rica	Switzerland	Nepal	France	The USA	Switzerland
Costa Rica	The USA	Nepal	Bangladesh	The USA	Spain
Costa Rica	Spain	Nepal	The UK	The USA	Kenya

Without mobility 95 (62%)					
The USA (16)		Egypt (2)		Iceland	
Germany (7)		Myanmar (2)		India	
Colombia (6)		Netherlands (2)		Malawi	
Australia (4)		Norway (2)		Mexico	
Nepal (4)		Pakistan (2)		New Zealand	
France (3)		Serbia (2)		Paraguay	
Peru (3)		Viet Nam (2)		Philippines	
Spain (3)		Austria		Poland	
The UK (3)		Belgium		Portugal	
Albania (2)		Chile		South Africa	
Bahamas (2)		Costa Rica		Sweden	
Bangladesh (2)		El Salvador		Thailand	
Bolivia (2)		Ethiopia		Trinidad and Tobago	
Brazil (2)		Guatemala		Uganda	

### 3.6

**APPENDIX 8:** Employers of PHPs employed, by sector (*N* = 152)

Academia *N* = 29 (19%)	
Aga khan University	Universidad Santo Tomás
Barcelona Global Health Institute	Universidad Surcolombiana
Bolivian Catholic University	University of Applied and Environmental Sciences
Carlos III Institute of Health	University of Birmingham
Escuela Militar de Ingeniería	University of California San Diego
German Diabetes Centre	University of Colorado
Institute for Health Metrics and Evaluation	University of Copenhagen
London School of Economics and Political Science	University of Hertfordshire
North South University, Bangladesh	University of Hohenheim
Sheffield Hallam University	University of Manitoba
Universidad de los Andes	University of Maryland
Universidad de Sonsonate	University of Nottingham
Universidad del Valle de Guatemala	University of Saskatchewan
Universidad Peruana Cayetano Heredia	University of York

Business, industrial, or commercial firm *N* = 23 (15%)	
Accenture	Janssen Cilag
ActiWay	Johns Hopkins University Applied Physics Laboratory
Alcon Vision LLC	Johnson&Johnson
Boston Scientific	Mercer
Cemka	MERCK SHARP & dohme
DJOGlobal	Mott Macdonald
Ecolab	Roche Diabetes Care
Hagerty Consulting	Sanofi
HERD International	VESO
IQVIA	

Government *N* = 29 (19%)	
Caribbean Public Health Agency	Österreichische Gesundheitskasse
City of Millcreek	Øygarden kommune
Consorci de Salut i Social de Catalunya	Public Health England
French Agency for Food, Environmental and Occupational Health & Safety	Santé publique France
French National Institute of Health and Medical Research	Sciensano
Health Protection New South Wales	The Bahamas Environment Science and Technology Commission
Human Fertilisation and Embryology Authority	The Robert Koch Institute
Ministry of health Paraguay	The USA Centres for Disease Control and Prevention
Ministry of Health of New Zealand	The USA Department of Health and Human Services
Ministry of Health of Brazil	Utah Department of Health
Ministry of Health, Wellbeing and Sports of The Netherlands	Victorian department of health and human services
National Institute for Health and Care Excellence	Washington State Department of Health
New South Wales Health	

Healthcare *N* = 24 (16%)	
National Health Services	People's Primary Healthcare Initiative (PPHI) Sindh
Clinic Nueva Rafael Uribe de Cali	Rehabilitation in the home
Clinic St. Pirminsberg, Canton St. Gallen, Switzerland	Rotherham National Health Services
Conselleria Sanitat Valenciana	Sanatorium Anchorena
E‐DA Healthcare Group	Sanitas
Genetic counselling centre	Sistema Unico de Saude
Institut national de la santé et de la recherche médicale	The Health Care Institution of South Iceland
Institute of Orthopaedic Surgery “Banjica”	Uganda Cancer institute
Molina Healthcare	University hospital Cologne
National Healthcare system of Panama	University of North Carolina Health Care System
Oxford University Clinical Research Unit‐Nepal	

Non‐governmental organisation *N* = 31 (20%)	
Save the Children International	Jhpiego
Action Against hunger	Kansas Democratic Party
American Cancer Society	Medical Action Myanmar
Centre for Care Innovations	Mental Health America of Georgia
Centre for Healthcare Improvement Research Social Company Limited	Première Urgence Internationale
Centre for European Policy	Programme for Appropriate Technology in Health
Child Health Research Foundation	Development Fund and Peruvian Branch of the Fulbright Commission
Comité d'educacion pour la sante de l’ herault	The International Committee of the Red Cross
Danish Red Cross	University of North Carolina Project
Doctors without borders	Vital Voices Costa Rica
Fundación Cristo vive	Winrock International
Group for Technical Assistance	World Association of Girl Guides and Girl Scouts

Intergovernmental organisation *N* = 12 (8%)	
Joint United Nations Programme on HIV/AIDS	United Nations Agency
Organisation for Economic Cooperation and Development	United Nations International Children's Emergency Fund
The Citizen Security and Justice Programme	United Nations Population Fund
The South Centre	World Health Organisation
The UN Refugee Agency	

Self‐employed 4 (3%)	
Director	Consultant in Public Health, Gender, and International Cooperation
Medical writer	Owner and founder

### 3.7

**APPENDIX 9:** Job titles of PHPs employed, per sector (*N* = 152)

Academia *N* = 29 (19%)		
Assistant professor	Health senior researcher	Project Manager
Associate professor	Lecturer	Research grant officer
Director of Epidemiology department	Professor	Vice‐dean
Educator	Programme coordinator	
External Relations Strategist	Project Leader	

Business, industrial, or commercial firm *N* = 23 (15%)		
Account Manager	Epidemiology Research Associate	Public health consultant
Analyst	Europe‐Middle East and Africa manager	Quality Analyst
Assistant Professor	Field Service Manager	Regional Labelling Liaison
Budget Project Manager	Global director	Research Fellow
Clinical Trial Contract	Health Economics & Market Access	Sales Manager
Consultant	Health economics and outcomes researcher	Senior Manager Associate
Data Management Officer	Healthcare technical advisor	Senior Regional Medical Manager
Deputy Project Manager	Junior quantitative researcher	Veterinarian sales and marketing responsible
Director	North American Language and Culture Assistant	
Epidemiologist	Policy adviser	
Epidemiology Manager for Latin American region	Professor	

Government *N* = 29 (19%)		
Biostatistician	Head of Research and Intelligence	Project researcher
Consultant	Health Promotion Officer	Public Health Advisor
Coordinator of studies and scientific support in epidemiology	Health Scientist	Public health officer
Data Services Manager	Health Services Consultant	Research assistant
Department technical advisor	Immunization Health Educator	Research Manager
Emergency Manager	Manager for health promotion	Scientific studies officer
Environmental therapist	Medical Officer	Senior Health Adviser
Epidemiologist	National Project Coordinator	Senior Policy officer
Epidemiologist and Evaluator	Phyto pharmacovigilance and Observatory of Pesticide Residues	Technical adviser

Healthcare *N* = 24 (16%)		
Chronic disease practitioner	Manager of Quality Interventions	Registered nurse
Community Nurse	Medical auditor	Research associate
Coordinator	Medical social worker	Researcher
Deputy Director Research	Nurse manager	Senior physician
Director of Pharmacy	Physician	Senior physiotherapist
Emergency attending	Preventive medicine and public health specialist	Speciality Doctor
Epidemiologist	Project Manager	Statistician
Healthcare quality coordinator	Public Engagement Officer	
Infectious Disease Specialist	Public Health Consultant	

Intergovernmental organisation *N* = 12 (8%)		
Chief of Health	Health Systems Consultant	Reproductive health officer
Consultant	Junior Policy Analyst	Sexual and Reproductive Health and Rights in Emergency Programme Specialist
Digital health consultant	Monitoring and Evaluation Specialist	Technical Consultant for COVID‐19 Response
Family Planning and Reproductive Health Commodity Security Adviser	Programme Officer	Technical Officer

Non‐governmental organisation *N* = 31 (20%)		
Community Outreach Manager	Health Officer	Programme Specialist
Deputy Chief of Party	Institutional advisor and regional coordinator	Project Coordinator
Deputy Director—Planning and Design, India Country Programme	Manager of Monitoring, Evaluation, Accountability and Learning Department	Project Medical Referent
Director of Nursing	Medical director	Researcher
Executive Director	Monitoring and Evaluation Assistant	Senior planning and Policy advisor
Field Programme Officer	Monitoring and Evaluation Officer	Senior Water, Sanitation and Hygiene Specialist and Adjunct Instructor
Functional Application Support Specialist	Operation manager	Study Coordinator
Health Advisor	Policy Analyst	Trauma Informed Systems Programme Manager
Health Coordinator	Programme Director	Water, sanitation and hygiene and public health expert
Health Expert	Programme Quality Officer	

Self‐employed 4 (3%).

### 3.8

**APPENDIX 10:** Job satisfaction of PHPs employed in health or public health—related employments using the Minnesota questionnaire‐short version (*n* = 147)

Question	Mean	SD
9. The chance to do things for other people (IF)	4.28	0.785
3. The chance to do different things from time to time (IF)	4.20	0.892
7. Being able to do things that do not go against my conscience (IF)	4.20	0.850
11. The chance to do something that makes use of my abilities (IF)	4.12	0.844
1. Being able to keep busy all the time (IF)	4.09	0.878
15. The freedom to use my own judgement (IF)	4.08	0.832
18. The way my co‐workers get along with each other	4.08	0.892
2. The chance to work alone on the job (IF)	4.04	0.882
4. The chance to be “somebody” in the community (IF)	4.04	0.856
16. The chance to try my own methods of doing the job (IF)	4.02	0.919
17. The working conditions	3.99	0.974
20. The feeling of accomplishment I get from the job (IF)	3.97	0.882
19. The praise I get for doing a good job (EF)	3.91	0.935
5. The way my boss handles his/her workers (EF)	3.84	0.994
8. The way my job provides for steady employment (IF)	3.84	1.120
6. The competence of my supervisor in making decisions (EF)	3.77	1.052
13. My pay and the amount of work I do (EF)	3.75	0.968
10. The chance to tell people what to do (IF)	3.74	0.798
12. The way company policies are put into practice (EF)	3.52	0.900
14. The chances for advancement on this job (EF)	3.52	1.207
General satisfaction (Maximum 100, minimum 43)	79.28/100	10.923
Intrinsic factors (Maximum 60, minimum 29)	48.64/60	6.509
Extrinsic factors (Maximum 30, minimum 10)	22.30/30	4.445

IF: Intrinsic factors, EF: extrinsic factors, all items count for general satisfaction.
